# Who Is Getting the Help They Need? An AI-Driven Study of Intersectional Disparities in Mental Health Service Utilization Among Young Adults with Suicidal Ideation

**DOI:** 10.1007/s11121-026-01924-0

**Published:** 2026-05-12

**Authors:** Hayoung K. Donnelly, Seonyeong Kim, Suna Kim, Eric S. Crosby, Maria A. Oquendo, Gregory K. Brown, Danielle L. Mowery

**Affiliations:** 1 Department of Psychiatry, University of Pennsylvania, Philadelphia, USA; 2 Institute for Biomedical Informatics, University of Pennsylvania, Philadelphia, USA; 3 Silver School of Social Work, New York University, New York, USA; 4 Department of Social Welfare, Seoul National University, Seoul, South Korea; 5 Department of Biostatistics, Epidemiology and Informatics, University of Pennsylvania, Philadelphia, USA

**Keywords:** Suicide prevention, Help-seeking behaviors, Mental healthcare utilization, Machine learning, Interpretable prediction model, Intersectionality

## Abstract

Despite the critical role of mental health services in suicide prevention, disparities in service utilization persist across various individual and social determinants of health. This study identifies key factors and intersectional patterns associated with mental healthcare use among young adults with past-year suicidal ideation, by developing machine learning-based artificial intelligence to improve predictive accuracy while ensuring interpretability for clinicians and policymakers. Utilizing cross-sectional data from the 2015–2020 National Survey on Drug Use and Health, we analyzed 11,018 US young adults aged 18–34 who reported past-year suicidal ideation. Random Forest and Shapley Additive Explanations identified the strongest predictors of mental healthcare utilization among 23 individual and social determinants of health. The decision tree model visualized prediction of utilization rates across intersectional characteristics. Findings indicate that depression, race/ethnicity, sexual orientation, and private health insurance were the strongest predictors of service use. Individuals without depression, males, Black, Indigenous, and People of Color, heterosexual individuals, and individuals without private health insurance were significantly less likely to seek care. These findings highlight the current intersectional disparities in mental healthcare utilization among young adults at risk of suicide. Expanding culturally competent care and promoting equitable access to mental healthcare for young adults at risk are crucial steps in addressing these disparities.

## Introduction

Suicide remains a major public health crisis in the USA and is the second leading cause of death among youth aged 10 to 18—a trend that extends into early adulthood (Ivey-Stephenson, 2022). While suicide deaths are a critical concern, the burden of nonfatal suicidal behaviors—including suicidal ideation, planning, and attempts—is substantially greater. Far more young adults experience these nonfatal behaviors, which often carry significant morbidity, making the prevention of suicidal ideation and attempts a key public health priority. Suicidal ideation is defined as thoughts about, considerations of, or plans for suicide (Klonsky et al., 2016). It exists on a spectrum, ranging from fleeting thoughts of death by suicide to a persistent preoccupation to kill oneself (Van Heeringen et al., 2000). Importantly, suicidal ideation is a strong predictor of suicide death and injury from suicide attempts, which are also associated with hospitalization, incarceration, and substantial financial costs (Nock et al., 2013; Shamsaei et al., 2020). Beyond its connection to suicide risk, prolonged suicidal ideation can profoundly disrupt developmental trajectories in young adulthood. Persistent suicidal thoughts may interfere with social, emotional, and cognitive growth, limiting opportunities for education, employment, and meaningful relationships (Czyz & King, 2015; De Luca et al., 2016).

### Individual Factors Associated with Mental Healthcare Utilization

Mental health services play a crucial role in reducing suicide risk among young adults experiencing suicidal ideation (Han et al., 2018a, 2018b). However, despite the availability of evidence-based treatments, many young adults do not seek or receive the care they need (Pottick et al., 2014). According to the Behavioral Model of Health Services Use (Andersen, 2005), individual determinants of healthcare utilization refer to an individual’s personal characteristics that influence their engagement with mental health services such as their race, ethnicity, and/or sexual identity (Fleury et al., 2014). Research consistently demonstrates that mental health service utilization varies by these individual determinants (Cook et al., 2017; Metzger et al., 2023). Black, Indigenous, and other People of Color (BIPOC) are significantly less likely than their White counterparts to seek mental healthcare (Green et al., 2020; Metzger et al., 2023). This disparity has been linked to historical and systemic factors, such as medical mistrust, negative experiences with healthcare providers, and limited access to culturally competent care (Goetz et al., 2023; Lam et al., 2022; Metzger et al., 2023). Sexual orientation is another factor associated with mental health service utilization, though findings remain mixed (Ferlatte et al., 2019). Some research suggests that LGBTQ+ young adults are more likely to seek mental health services, but not often from family or community members (Baams et al., 2018). However, other studies indicate that barriers such as stigma, discrimination, and a lack of affirming providers may deter service use (Dunbar et al., 2017). As such, it is crucial to recognize that young adults experiencing suicidal ideation are not a uniform group for addressing disparities in mental health service use.

### Social Factors Associated with Mental Healthcare Utilization

Alongside individual factors, social and environmental conditions also significantly impact young adults’ access to and utilization of mental health services. According to the social determinants of health framework (SDoH; Kirkbride et al., 2024), social determinants of healthcare utilization refer to societal characteristics that individuals are exposed to across the lifespan that affect their healthcare use such as income, employment, socioeconomic status (Kirkbride et al., 2024). The SDoH framework provides a valuable perspective for understanding how these broader social, economic, and environmental conditions shape healthcare use (Alegria et al., 2018; Kirkbride et al., 2024). A systematic review of barriers to mental health service utilization found that socioeconomic status is an important factor, with lower-income individuals often experiencing greater barriers to accessing care (Williams et al., 2019). Insurance status has also been linked to service utilization, with studies indicating that young adults covered by Medicaid, no insurance, or alternative insurance plans are less likely to seek mental health services compared to those with private insurance (Pottick et al., 2014). Young adults experiencing housing instability or classified as not in education, employment, or training (NEET) are at heightened risk for mental health challenges and are less likely to seek formal care (Filia et al., 2022). In addition, geographic variation, particularly urbanicity, has been associated with disparities in access, as individuals in rural or underserved areas may encounter a shortage of mental health providers, limiting available care options (Kim et al., 2017).

### Intersectionality and Mental Healthcare Utilization

Individual and social factors interact in complex ways, shaping mental health service utilization. According to intersectionality theory, as originally articulated by Crenshaw (1989), individuals experience overlapping systems of privilege and oppression that influence their help-seeking behaviors. Empirical evidence illustrates how intersecting social identities jointly, such as race/ethnicity and sexual orientation, contribute to further disparities in mental healthcare utilization. Recent population-based analyses demonstrate that sex, and race/ethnicity interact to shape patterns of mental health service use, and socioeconomic conditions such as insurance status further modify these inequities (Olisaeloka et al., 2025). Extending this pattern, Black bisexual individuals, non-Hispanic Asian gay/lesbian individuals, and Hispanic/Latino sexual minorities face greater barriers to care compared to White heterosexual individuals (Turpin et al., 2021). Similarly, studies of sexual minority populations indicate that structural barriers such as cost, provider availability, and geographic access, interact with minority stress processes that deter help-seeking (Cronin et al., 2021a, b). The intersection of individual factors and SDoH is evident in the structural barriers faced by sexual and gender minorities in rural areas, where limited access to LGBTQ-affirming providers and long travel distances further restrict their ability to seek mental healthcare (Joudeh et al., 2021). Recent quantitative health research highlighted that intersectionality risks becoming diluted when treated as a descriptive label for demographic heterogeneity rather than as a framework guiding analytic design (Guan et al., 2021). Collectively, these findings underscore the importance of an intersectional approach in addressing disparities through targeted outreach, culturally competent care, and structural interventions to reduce inequities among marginalized populations.

Although the decisions to seek mental healthcare are influenced by multiple factors and their interactions as discussed above, some studies have primarily focused on one or a few demographic factors to explain mental healthcare use, rather than considering multiple predictors in their research model (Roberts et al., 2018). Most studies on intersectionality and health equity have focused primarily on intersections of sex/gender and race/ethnicity (Harari & Lee, 2021). This approach may overlook or misidentify the most influential predictors of mental healthcare use, limiting the development of effective strategies to identify target populations for interventions to increase mental healthcare utilization. It was recommended to incorporate additional social characteristics (e.g., sexual orientation, religion, and age) beyond these two categories when examining intersectionality and health equity (Harari & Lee, 2021). In contrast, some studies have established that mental healthcare utilization is shaped by the intersection of multiple individual characteristics and social determinants of health, and these studies have accordingly incorporated multiple predictors within intersectional analytic frameworks to better understand disparities in help-seeking behaviors (Cronin et al., 2021a, b; Turpin et al., 2021). However, as relevant predictors become more numerous and interrelated, conventional analytic approaches may struggle to simultaneously assess their relative predictive importance, account for nonlinear associations, and capture complex interactions at scale (Bzdok & Meyer-Lindenberg, 2018).

### Computational Methods for Understanding Mental Healthcare Utilization

The application of machine learning in this study aims to supplement traditional multivariate approaches by offering additional advantages. For instance, traditional statistical models often focus on validating predefined relationships as a confirmatory approach, such as hypothesis testing, to explain the associations between factors and outcomes (Bzdok & Meyer-Lindenberg, 2018). However, machine learning is, by nature, data-driven and exploratory, allowing it to embrace the potential of diverse predictors to improve outcome prediction. Statistical multivariate approaches, such as latent class and profile analyses, identify behavioral patterns and classify individuals based on multiple characteristics, but they do not inherently optimize predictive accuracy for outcomes such as service utilization. Machine learning also allows researchers to comprehensively evaluate and validate predictive performance of multiple features across different models, rather than relying on a single model. While traditional approaches draw conclusions from a one-step analysis (e.g., using the entire dataset to answer a research question), machine learning often uses a two-step process such as the hold-out method (Bzdok & Meyer-Lindenberg, 2018). For example, the hold-out method splits the dataset into two subsets (e.g., training and test sets), using one subset to train the model and the other to evaluate its performance. Another validation method in machine learning involves validating findings across different algorithms (e.g., decision tree, logistic regression; Donnelly et al., 2023). By using cross-validation, machine learning approaches also provide findings that are repeatedly validated, both in terms of outcome prediction and the importance of features, offering a more robust assessment compared with traditional research models (Bzdok & Meyer-Lindenberg, 2018).

Another methodological priority of the current study is to provide interpretable findings from machine learning models, in addition to evaluating models’ accuracy. Interpretability and explainability of artificial intelligence (AI) are as crucial as predictive accuracy in mental healthcare research, particularly when the goal is to provide actionable insights for clinicians and policymakers. Widely used interpretable machine learning approaches include SHAP (Shapley Additive Explanations) and decision trees (Molnar, 2020). For instance, decision tree provides a sequential visualization of how different factors relate to the target outcome by showing the hierarchical prediction process within a single figure (De Ville, 2013; Good et al., 2023). Incorporating these techniques makes the model easier to understand and apply in discussions of real-world suicide prevention efforts, going beyond evaluating machine learning solely for its high predictive performance.

This study aims to provide evidence on where disparities in mental healthcare use exist among young adults with suicidal ideation and to discuss strategies for promoting healthcare utilization among them, incorporating a computational and data-driven approach. It identifies key factors that are relatively more important in predicting mental healthcare use among young adults with suicidal ideation. The intersectionality between individual and social determinants of health and its relation to mental healthcare utilization among young adults with suicidality is also illustrated. This study also focuses on an explainable AI approach to deliver interpretable findings for clinicians and policymakers, increasing the utility and adoptability of machine learning models in clinical settings. Our research questions include (1) Which factors most strongly predict mental healthcare utilization among young adults with suicidal ideation? and (2) How do these factors intersect to shape disparities in mental health service utilization among young adults with suicidal ideation?

## Methods

### Data Source and Participants

The study analyzed cross-sectional data from the 2015–2020 National Survey on Drug Use and Health (NSDUH). NSDUH is an annual, population-based survey administered in the USA by the Substance Abuse and Mental Health Services Administration (SAMHSA) to assess various aspects of substance use, mental health, and healthcare services. NSDUH employs a multistage area probability sampling design with multiple levels of stratification, including states and state sampling regions (SSRs). Data collection methodology has been previously reported for each year (Center for Behavioral Health Statistics & Quality, 2022). The sample weights provided by NSDUH were not incorporated into the machine learning model development. Our approach aligns with prior machine learning applications (Si et al., 2025), takes into account the random sampling assumption underlying most machine learning algorithms (Si et al., 2025), and accounts for the limited availability of machine learning software supporting complex survey designs (MacNell et al., 2023).

The initial dataset included 127,589 young adults aged 18 to 34. We included young adults who endorsed suicidal ideation (i.e., *At any time in the past 12 months, did you seriously think about trying to kill yourself?*) and reported a valid response for inpatient or outpatient mental health service use, or mental health medication use in the past year (see Measures section below). Therefore, the final sample included 11,018 young adults, all of whom reported having suicidal ideation and provided information on whether they used mental healthcare in the past year.

### Measures

We developed a machine learning model to predict mental healthcare utilization using 23 individual and social determinants of health as predictors. Measures aligned with NSDUH methodology terms and definitions; however, some coding adjustments were made to maintain consistency in variable scaling and standardization.

#### Mental Healthcare Utilization

The primary outcome variable was past-year mental health service use, which was examined using three yes/no questions. Participants were asked about past-year inpatient mental health service use (i.e., *During the past 12 months, have you stayed overnight or longer in a hospital or other facility to receive treatment or counseling for any problem you were having with your emotions, nerves, or mental health?*), outpatient mental health service use (i.e., *During the past 12 months, did you receive any outpatient treatment or counseling for any problem you were having with your emotions, nerves, or mental health?*), and mental health medication use (i.e., *During the past 12 months, did you take any prescription medication prescribed to treat a mental or emotional condition?*). If participants responded “Yes” to at least one question, they were coded as having used any mental healthcare service (1 = Yes), and those who responded “No” to all the questions were coded as not using any mental healthcare service (0 = No).

#### Demographics and Clinical Characteristics

Demographic and clinical predictor variables included sex, age, race/ethnicity, sexual orientation, marital status, English fluency, religiosity, overall health, depression status, substance dependence, and timing of survey completion. Sex was coded as 1 = Male and 2 = Female. Age was measured on an ordinal scale (1 = 18 years old, 2 = 19 years old, 3 = 20 years old, 4 = 21 years old, 5 = 22 or 23 years old, 6 = 24 or 25 years old, 7 = 26 to 29 years old, and 8 = 30 to 34 years old). Race/ethnicity was coded as 1 = Non-Hispanic White, 2= Non-Hispanic Black/African American, 3 = Hispanic, 4 = Non-Hispanic Asian American/Pacific Islander, 5 = Non-Hispanic Native American/Alaska Native, and 6 = Non-Hispanic More Than One Race. Sexual orientation was coded as 0 = Heterosexual and 1 = Lesbian, Gay, or Bisexual (LGB). Marital status was categorized as 1 = Married, 2 = Widowed, 3 = Divorced or Separated, and 4 = Never Married. English fluency was assessed using the question *How well do you speak English?* with responses ranging from 1 = Very well to 4 = Not at all. Religiosity was assessed with the statement, *Your religious beliefs are a very important part of your life*, with responses coded from 1 = strongly disagree to 4 = strongly agree. The year of data collection was coded as 1 = 2015, 2 = 2016, 3 = 2017, 4 = 2018, 5 = 2019, 6 = 2020.

Overall health was assessed on a scale from 1 = excellent to 5 = poor. Depression status was determined based on whether an individual experienced at least five symptoms of a major depressive episode for at least two weeks within the past 12 months. Those meeting this criterion were coded as 1 = Yes, while those who did not were coded as 0 = No. The Nicotine Dependence Syndrome Scale and the Fagerstrom Test of Nicotine Dependence assessed past-month nicotine dependence. Those classified as nicotine dependent on either scale were coded as 1 = Nicotine Dependent, while others were classified as 0 = No Nicotine Dependence. Alcohol, illicit drug, and psychotherapeutic drug dependence were determined based on whether an individual experienced at least three symptoms of substance dependence listed in the 4th or 5th Edition of the Diagnostic and Statistical Manual of Mental Disorders (DSM) in the past 12 months.

#### Social Determinants of Health

Social determinants of health predictor variables included household income, poverty level, urbanicity, NEET status, high school educational attainment, and health insurance type. Household income was categorized as 1 = Less than $20,000, 2 = $20,000–$49,999, 3 = $50,000–$74,999, and 4 = $75,000 or more. Poverty levels were defined based on the US Census Bureau’s poverty threshold by family unit size. Young adults were classified as living in poverty if their family income fell below this threshold and were classified as not living in poverty if their family income met or exceeded the threshold (1 = Living in Poverty; 0 = Not Living in Poverty). Urbanicity of the county where participants resided was coded as 1 = Large Metro, 2 = Small Metro, 3 = Non-metro based on the US Department of Agriculture’s (USDA) Rural–Urban Continuum Codes. For the NEET—Not in education, employment, or training—status, individuals who reported not currently attending any school and had not worked at any job in the past 12 months were coded as 1 = NEET youth, while all others were coded as 0 = Non-NEET youth. High school educational attainment was coded as 1 if respondents had a high school diploma, GED, or an equivalent degree, and were coded 0 if they did not meet any of these criteria. In terms of insurance type, young adults were asked whether they were covered by any of four types of insurance: Medicaid, Medicare, Military and Veteran insurance, or private health insurance. Respondents were coded as 1 = Yes if they had any of the four types of insurance and 2 = No if they had none of the four.

### Machine Learning Approaches

First, we developed a Random Forest model and applied the Tree SHAP algorithm to identify the most important factors predicting mental healthcare utilization. Second, we constructed a decision tree using the key predictors identified in the first step to examine how individual or combined factors relate to mental healthcare utilization. This study aims to explore intersectionality using findings from a decision tree, while Random Forest and SHAP were applied as a feature selection step to assess each factor’s importance before constructing the decision tree. The Python packages *pandas* and *numpy* were used for data integration and feature engineering, *sklearn* was used for implementing the Random Forest algorithm, *shap* was used for SHAP value analysis, and *ctree* in R was used to construct the decision tree.

Random Forest is well-suited for predicting binary outcomes from high-dimensional data and reducing bias as a tree ensemble model (Breiman, 2001; Hu & Szymczak, 2023; Wang et al., 2024). It is particularly known for its ability to rank features according to their contribution to outcome predictions, a property referred to as feature importance. This makes it one of the most widely used approaches for feature selection (Wang et al., 2024). We focused on Random Forest rather than comparing multiple algorithms or exploring different ensemble methods. By focusing on a single method, we aim to provide an in-depth analysis of the factors themselves that are related to mental health service use, while maintaining adequate model performance. Random Forest often outperforms alternative machine learning methods across both literature review and experimental studies involving various datasets (Fawagreh et al., 2014; Hu & Szymczak, 2023).

The Random Forest model in this study was developed using tenfold cross-validation and 70:30 hold-out method, with 70% of the data used to train the model and 30% to test it. We used stratified sampling to ensure that the training and testing subsets maintained the same distribution of the outcome variable as the entire sample during these validation processes. In other words, each subset included positive cases (those who used mental health services) and negative cases (those who did not), with proportions matched to those of the overall dataset. A grid search was conducted to fine-tune the model by comparing different hyperparameters, including the number of trees (*ntree*) [500, 1000, 1500], the number of features considered at each split (*mtry*) [‘auto’, ‘sqrt’, ‘log2’], the maximum tree depth [5, 10, 15], and the threshold. Model performance was evaluated using the Area Under the Curve (AUC) of the Receiver Operating Characteristic (ROC), sensitivity, specificity, positive predictive value, and negative predictive value. Importance scores were normalized on a scale ranging from 0 (least important factor) to 1 (most important factor) and ranked to facilitate comparison. Factors above the overall average importance across factors were considered key predictors of mental healthcare utilization.

The Tree SHAP (Shapley Additive Explanations) algorithm was applied to validate the Random Forest results. SHAP is another widely used feature selection method and is also considered an interpretable machine learning algorithm (Molnar, 2020). Both Random Forest and SHAP provide information on how strongly each feature is related to the prediction of outcomes. While Random Forest feature importance is derived automatically from the trained trees, SHAP computes the contribution of each feature to predictions as an independent step alongside model training (Wang et al., 2024). Examining Shapley values of features can help validate the feature importance derived from Random Forest to confirm the most important predictors. For instance, SHAP has often been used in combination with Random Forest or as a post-hoc analysis to further understand Random Forest’s predictions (Sarica et al., 2023; Song et al., 2025; Taye et al., 2025). A higher Shapley value indicates greater importance of a factor. Factors with scores exceeding the overall average in Shapley values were considered key predictors of mental healthcare utilization. The final set of key predictors was selected based on the highest-ranking factors across both the Random Forest and SHAP models.

A decision tree was constructed using these key predictors to examine their intersectional relationships with healthcare utilization and to provide an interpretable representation of the prediction process. Decision tree methods have been used in previous studies to incorporate the intersectionality framework in the development of their prediction models (Mahendran et al., 2022; Mena et al., 2021; Varga et al., 2026). Although the decision tree may not estimate statistical interactions and linear relationships in the classical sense, it captures the joint contribution of multiple features in prediction (Molnar, 2020). In a decision tree, the predicted outcome at a terminal node (e.g., probabilities of mental healthcare use) is based on the path from the root to that node, which represents a specific combination of feature conditions (Molnar, 2020). The conditional probabilities at the terminal nodes, with multiple factors, provide insights into whether specific intersectional subgroups are more or less likely to use mental health services. This approach therefore provides insights into the intersectional characteristics of subgroups in relation to mental healthcare use. Furthermore, the decision tree provides an intuitive, interpretable way to track trajectories of different factors, helping visually understand how combinations of these factors influence the probability of mental health service use (Molnar, 2020).

## Results

The final analytic sample consisted of 11,018 young adults aged 18–34 who reported past-year suicidal ideation and provided valid information on mental healthcare utilization. The average age of young adults was 23.5 years. The majority identified as female (58.9%) and White (59.8%). Other racial/ethnic identities included Black/African American (10.3%), Hispanic (17.2%), Asian American/Pacific Islander (4.3%), Native American/Alaska Native (1.7%), and multiracial (6.7%). Regarding sexual orientation, 71.6% identified as heterosexual, while 28.4% identified as being lesbian, gay, or bisexual. Out of 11,018 young adults with suicidal ideation, 44.7% reported using mental health services at least once in the past year, while 55.3% reported no mental health service utilization.

### Key Predictors of Mental Healthcare Utilization

**Fig. 1:**
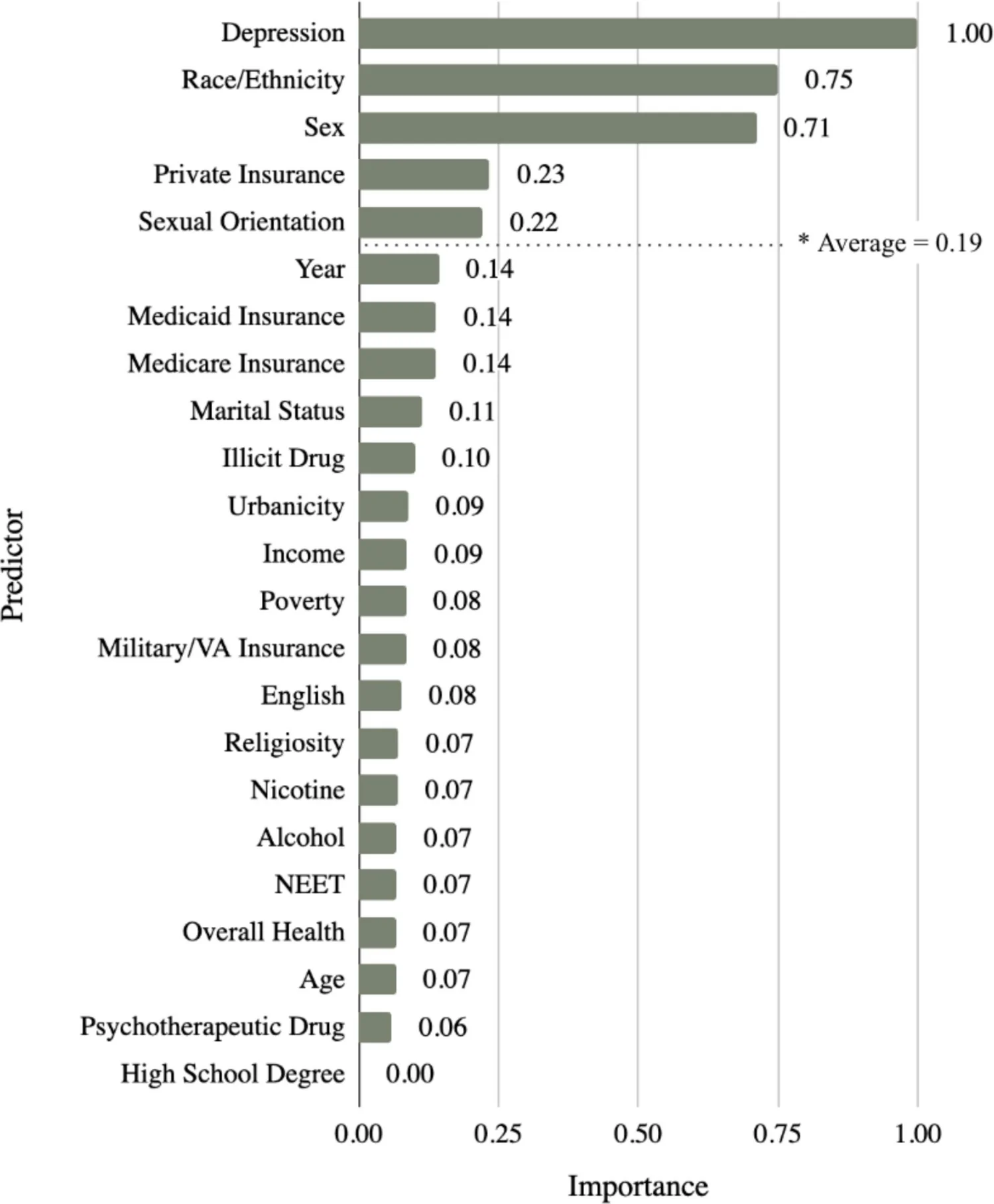
Factor importance for predicting mental healthcare use based on Random Forest

The Random Forest model demonstrated good or excellent performance (i.e., AUC > 0.80; White et al., 2023) in predicting mental healthcare utilization among young adults with suicidal ideation in the past year. As a result of the grid search, the final hyperparameters were set as follows: ntree = 500, mtry = sqrt, maximum depth of the tree = 10, and the threshold was recommended to be 0.46. The prediction results showed an AUC of 0.85 for the ROC curve, with sensitivity of 0.77, specificity of 0.77, positive predictive value of 0.72, and negative predictive value of 0.80. The average importance of the 23 factors in predicting service utilization was 0.19 (Fig. 1). Five predictors—depression (Importance score = 1), race/ethnicity (Importance score = 0.75), sex (Importance score = 0.71), private insurance status (Importance score = 0.23), and sexual orientation (Importance score = 0.22)—were key predictors, with importance levels higher than the average.

**Fig. 2:**
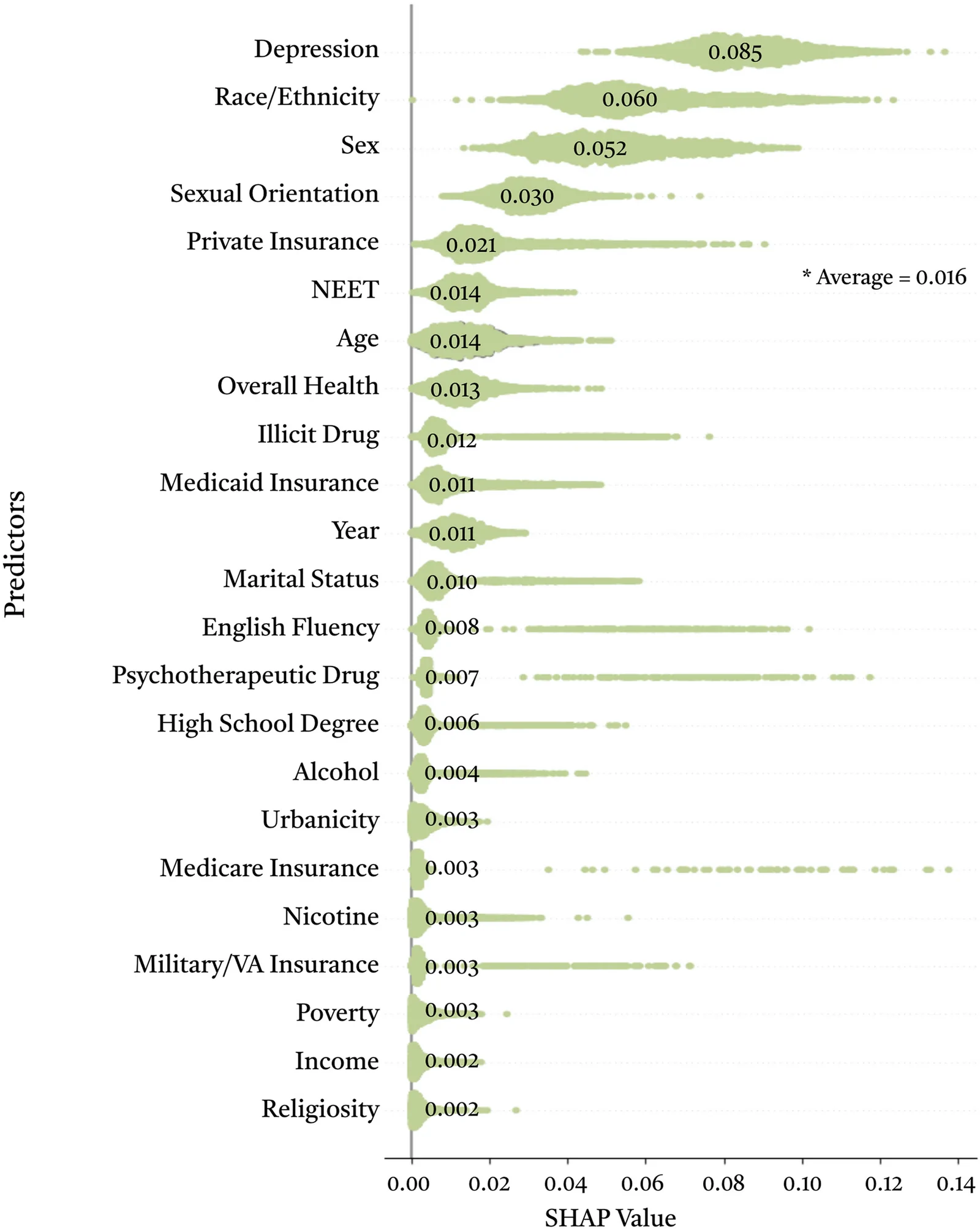
Factor importance for predicting mental healthcare use based on SHAP

The average Shapley value across the 23 predictors was 0.016 (Fig. 2). Consistent with the Random Forest results, five factors—depression (Shapley value = 0.085), race/ethnicity (Shapley value = 0.060), sex (Shapley value = 0.052), sexual orientation (Shapley value = 0.030), and private insurance status (Shapley value = 0.021)—were identified as key predictors, with values higher than this average.

### Prediction Pathway

**Fig. 3:**
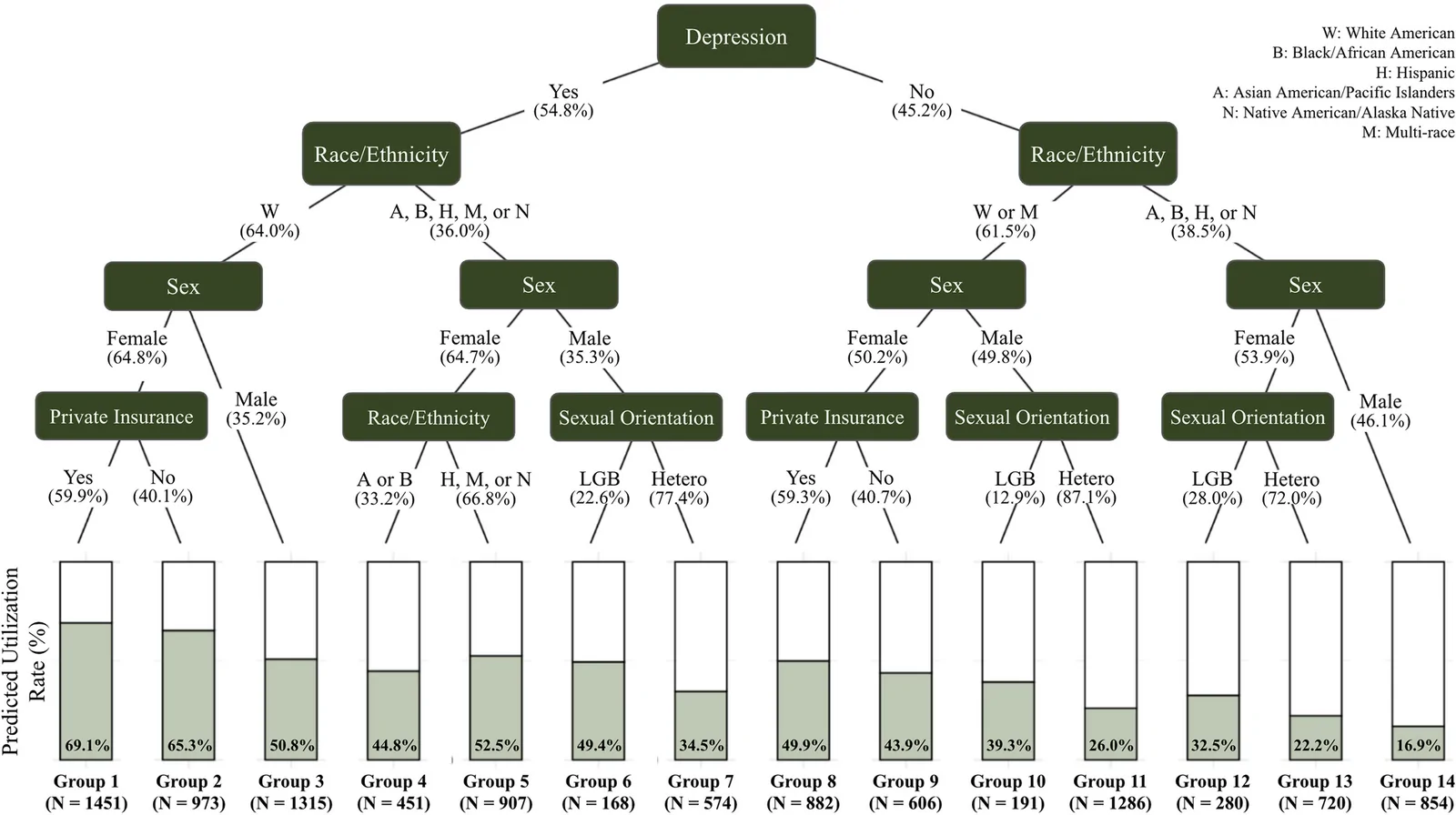
Prediction pathway of the likelihood of mental healthcare use among young adults with suicidal ideation

Using the key predictors of mental healthcare service use—depression, race/ethnicity, sex, sexual orientation, and private insurance status—the decision tree identified 14 distinct groups of young adults with past-year suicidal ideation, categorized based on their likelihood of utilizing mental health services (Fig. 3). Group 1 was predicted to be the most likely to use mental healthcare services, while Group 14 was predicted to be the least likely. Group 1 (N = 1451) was young adults with past-year suicidal ideation who had depression, identified as White and female, and had private insurance. They were expected to use mental healthcare services 69.1% of the time when experiencing suicidal thoughts. Group 14 (N = 854) was young adults with past-year suicidal ideation who did not have depression, identified as either Asian, Black, Hispanic, or Native American/Alaska Native, and were male. They were expected to use mental health services 16.9% of the time when they experienced suicidal thoughts.

Depression, race/ethnicity, and sex were consistent key predictors of service utilization across all groups. The likelihood of young adults with depression (Groups 1–7) using mental health services was 34.5% (Group 7) to 69.1% (Group 1), while the likelihood of young adults without depression (Groups 8–14) using mental health services was between 16.9% (Group 14) and 49.9% (Group 8). Across racial/ethnic groups, White young adults were the most likely to use mental healthcare services, while Black/African American and Asian American/Pacific Islanders were the least likely to use them. White young adults had a 26.0% (Group 11) to 69.1% (Group 1) chance of using mental health services, while Black/African American and Asian American/Pacific Islander young adults had a 16.9% (Group 14) to 49.4% (Group 6) chance of using mental health services. The likelihood of Hispanic and Native American/Alaska Native young adults using mental health services ranged from 16.9% (Group 14) to 52.5% (Group 5). The likelihood of multi-racial young adults using mental health services ranged from 26.0% (Group 11) to 52.5% (Group 5). The likelihood of using mental health services ranged from 22.2% (Group 13) to 69.1% (Group 1) among female young adults and from 16.9% (Group 14) to 50.8% (Group 3) among male young adults.

Private insurance and sexual orientation were unique key predictors of service utilization for specific subgroups (i.e., Group 1, 2, 6, 7, 8, 9, 10, 11, 12, 13). For private insurance, among young adults with depression who are White and female, those with private insurance (Group 1) were predicted to be 3.8% more likely to use mental health services than those without private insurance (Group 2). Among young adults without depression who are either White or multi-racial and female, those with private insurance (Group 8) were predicted to be 6% more likely to use mental health services than those without private insurance (Group 9). For sexual orientation, among young adults with depression who are non-White and male, those who identified as LGB (Group 6) were predicted to be 14.9% more likely to use mental health services than heterosexual young adults (Group 7). Among young adults without depression who are either White or multi-racial and male, those who identified as LGB (Group 10) were predicted to be 13.3% more likely to use mental health services than heterosexual young adults (Group 11). Among young adults without depression who are either Asian American/Pacific Islander, Black/African American, Hispanic, or Native American/Alaska Native and female, those who also identified as LGB (Group 12) were predicted to be 10.3% more likely to use mental health services than heterosexual young adults (Group 13).

## Discussion

Identifying which young adults with suicidal ideation tend to not seek mental health services is vital for preventing suicidal behavior. The current study examined the association between both individual and societal characteristics that determine mental healthcare use, and their interaction on mental health service utilization among a nationally representative sample of young adults with past-year suicidal ideation using a machine learning algorithm. Our findings showed that the most robust positive predictors of mental health service use among young adults with past-year suicidal ideation were depression, racial/ethnic identity, sex, sexual orientation, and private health insurance status.

Young adults with past-year suicidal ideation who did not experience a major depressive episode were less likely to seek mental health services than those who did experience a major depressive episode. This aligns with previous research that found young adults with suicidal ideation were less likely to seek treatment compared to those with depression (Cheung & Dewa, 2007). This finding may be due to a lack of perceived need for treatment or maladaptive coping strategies (Hom et al., 2015). Young adults with suicidal ideation frequently report that their stress is normal and express uncertainty about the seriousness of their mental health needs (Downs & Eisenberg et al., 2012). In addition, suicidal ideation is associated with decreased problem-solving ability among young adults (Heapy et al., 2024), and poor problem-solving ability is associated with decreased openness towards mental health help-seeking (Segal et al., 2014). On the other hand, young adults with both suicidal ideation and depression may have greater need for mental health services due to a greater number of symptoms and more severe functional impairments. Once these young adults initiate mental health treatment, they may receive additional knowledge about mental health symptoms and be provided encouraging stories about the positive effects of mental health treatment that help sustain treatment engagement (Eigenhuis et al., 2021). These findings underscore the need for routine suicide screening and assessment to raise awareness of suicide risk and the benefits of treatment. This may increase perceived need for treatment and engagement in mental health services as an adaptive coping strategy.

Consistent with racial/ethnic differences in mental health services use in the USA, BIPOC with past-year suicidal ideation used mental health services less than White individuals with past-year suicidal ideation (Lê Cook et al., 2017). This disparity may be, in part, due to a lack of trust in the healthcare system and socioeconomic inequalities experienced by minoritized racial/ethnic groups. For example, non-treatment-seeking Asian and Black young adults with past-year suicidal ideation were more likely to cite cultural insensitivity from mental health providers as a barrier to treatment than White young adults (Samlan et al., 2021). In addition, non-treatment-seeking Hispanic young adults with past-year suicidal ideation were more likely to report finances as a barrier to mental healthcare than their White counterparts (Samlan et al., 2021). Strategies to improve mental healthcare access for BIPOC young adults with suicidal ideation align with broader efforts to expand healthcare access for minoritized populations. It is crucial to provide mental health education to reduce stigma to young adults with suicidal ideation and connect them to mental health agencies that maintain a diverse workforce, offer culturally responsive care, and provide various flexible payment options (George et al., 2025; Goetz et al., 2023; Planey et al., 2019).

Regardless of depression status or race, males with past-year ideation were less likely to use mental health services than females with past-year ideation. This finding aligns with the sex differences in mental service use among young adults (Eisenberg et al., 2011). Young adult males with past-year suicidal ideation may be less likely to use mental health services than their female counterparts due to their negative attitudes toward mental health help-seeking. Male young adults with past-year suicidal ideation are more likely to report that they prefer to manage their mental health issues on their own and report that they do not have a need for mental health services than female young adults with past-year suicidal ideation (Samlan et al., 2021). This may be due to stigma, as some men fear being perceived as weak by their peers for seeking mental health treatment (Lynch et al., 2018). Nonjudgmental peer support can help normalize suicidal ideation and its associated distress and functional impairments among young adult males (Sagar-Ouriaghli et al., 2019). This may include male peers with suicidal ideation identifying signs of elevated suicide risk, providing on-demand emotional support for acute crises, and assisting in triage to professional mental health services (Bowersox et al., 2021). These peer support services may make engagement in professional mental health services more acceptable for young adults with suicidal ideation.

Heterosexual young adults with past-year ideation were less likely to use mental healthcare services than Lesbian, Gay, and Bisexual (LGB) young adults with past-year ideation, similar to help-seeking rates by sexual orientation among young adults without suicidal ideation (Baams et al., 2018). LGB young adults may use mental health services more frequently than heterosexual individuals due to more frequent experiences of discrimination and a greater prevalence of mental health symptoms (Burgess et al., 2007). This suggests that differences in mental health treatment engagement by sexual orientation may be driven by the unique life circumstances and mental health needs of each group. To better understand these differences, future research should explore the factors that predict mental health service use in both LGB and heterosexual populations. Identifying the life circumstances and individual characteristics associated with mental health service use can help develop targeted strategies to improve access to mental healthcare and ensure that services effectively meet the needs of each population (Chang et al., 2025).

Private health insurance in the USA is largely concentrated among individuals with stable jobs, higher wages, and employer-sponsored benefits. Wray et al. (2021) note that employer-sponsored insurance remains the most common form of private coverage, making it the largest single category by any measure. Private insurance holders typically experience fewer coverage lapses, access a broader range of healthcare providers, and encounter fewer barriers to specialized services compared to those relying on public insurance programs such as Medicaid or Medicare. However, these advantages are not equitably distributed across other characteristics, such as race/ethnicity. Populations with higher socioeconomic status, predominantly White, are more likely to have private insurance, whereas racial and ethnic minorities are disproportionately enrolled in public insurance programs or remain uninsured (Rodgers et al., 2022). Notably, people with private insurance often face fewer barriers to accessing mental healthcare than those with public insurance (Brahmbhatt & Schpero, 2024; Wiznia et al., 2017).

For individuals without private insurance, access to in-network mental health providers can be limited, and out-of-network mental healthcare can be prohibitively expensive. Though Medicaid is vital in providing coverage for low-income populations, it has critical limits, as enrollees often have difficulty accessing specialized mental health services because of provider shortages and low reimbursement rates, which discourage mental health professionals from accepting Medicaid patients (Zhu et al., 2021; Zhu et al., 2023). These restrictions are particularly concerning for individuals in crisis since those experiencing suicidality often refrain from seeking help due to financial constraints and limited service accessibility (Rastogi et al., 2024). For example, many low-income individuals relying on Medicaid work multiple jobs or shoulder caregiving responsibilities, making it difficult to find time for appointments even when services are covered (Ayanian et al., 2018; Eden & Schulz, 2016). Additionally, those who live in rural areas with a severe shortage of mental health providers would require significant travel commitments that further hinder access to care (Morales et al., 2020; Negaro et al., 2023). Even when Medicaid beneficiaries are theoretically covered, they often face longer wait times and reduced access to quality mental healthcare compared to those with private insurance (Brahmbhatt & Schpero, 2024; Wiznia et al., 2017). This highlights the need for policy interventions aimed at lowering costs and expanding financial assistance programs for low-income individuals, which could further enhance healthcare utilization (Hooley et al., 2022).

While it is important to consider each factor individually, understanding the intersectionality of these factors is crucial, as our decision tree demonstrated, to accurately identify the groups most in need of support and to develop tailored interventions. Individual characteristics such as race/ethnicity, gender, sexual orientation, and SDoH such as income and insurance do not operate in isolation but interact in complex ways to shape mental health service utilization. Prior research has shown that individuals with multiple marginalized identities, such as racial and sexual minorities, often face more barriers to accessing care and unique forms of social exclusion deterring them from seeking support (Joudeh et al., 2021; Turpin et al., 2021). Similarly, young adults experiencing multiple structural disadvantages, including housing instability, unemployment, and economic hardship, report significantly higher levels of psychological distress, depression, and suicidal ideation (Filia et al., 2022). The compounding effects of these disadvantages suggest that individuals experiencing multiple forms of social exclusion may face greater challenges in using mental healthcare services than those experiencing a single disadvantage. Expanding culturally and contextually competent care, improving affordability, and tackling structural barriers such as financial constraints and provider shortages are crucial. Additionally, efforts to reduce stigma and discrimination within healthcare systems must be prioritized to ensure that individuals from historically marginalized backgrounds receive the support they need. By adopting an intersectional framework, healthcare systems and policymakers can effectively identify high-risk groups and develop strategies that address the overlapping barriers preventing mental healthcare utilization.

To expand this study and validate its findings, future research should address the limitations and suggestions outlined in the current study. This study aimed to include as many predictors as possible known to be relevant to suicide risk and mental healthcare utilization. However, due to the limitation of using secondary data, certain factors could not be included. For example, the transgender community (Feldman et al., 2021; Vigny-Pau et al., 2021), individuals with a history of incarceration (Canada et al., 2022), and those transitioning from foster care to independent living (Katz et al., 2024) report a higher risk of suicide but limited mental healthcare service. However, this information was not provided from the original data source. Due to the cross-sectional nature of the data, it is important to note that the relationships between factors are statistically predictive, but do not guarantee causal effects. Applying longitudinal data and analytical techniques would be beneficial in examining whether the key predictors are causal factors, rather than merely correlated factors, in mental health utilization. Because the current dataset includes multiple years of cross-sectional survey data, it is possible that the same individuals participated in different yearly datasets. Since identifiers were not available, potential correlations within individuals could not be accounted for. Therefore, future studies could validate the current findings by ensuring independence of data through analyzing each year separately or by using panel data. To address potential temporal dependency from the use of data across multiple years in training and testing, methods such as time-based splitting or chronological cross-validation can be considered to account for temporal order (Bergmeir & Benítez, 2012; De Nicolò et al., 2024).

We employed a method that provides findings that are intuitive and interpretable for a broader audience who may not be familiar with machine learning or computational approaches. The research design with Random Forest was tailored to foster active discussion on improving healthcare service use among clinicians and policymakers by presenting results in an easily understandable way. While this study focuses on Random Forest and the model demonstrates fair performance (AUC, sensitivity, etc., all over 0.7), performance could be improved by applying different machine learning algorithms (e.g., Support Vector Machine, XGBoost) or using an ensemble model that combines multiple algorithms in a future study. Traditional statistical approaches for examining intersectionality, such as including interaction terms, testing moderators, or conducting subgroup analyses, may further help validate the findings from the decision tree. Since sampling weights were not incorporated in the machine learning models, the results may differ from analyses that use weighted data (Nalenz et al., 2024). Future research incorporating post-hoc adjustments will be needed to address this limitation. For instance, approaches such as Hájek-forests, which have been shown to effectively accommodate complex health data with unequal probability sampling, can be considered (Nalenz et al., 2024).

Mental healthcare utilization was operationalized as any mental healthcare use in the past year, combining multiple treatment types, settings, and care models. While this approach is consistent with prior health services research focused on engagement with care (Fleury et al., 2014), it may obscure heterogeneity in treatment pathways. As such, findings should be interpreted as reflecting factors associated with any mental healthcare engagement, rather than treatment utilization patterns. Therefore, future models should consider additional potential predictors with modality-specific analyses to disentangle the process underlying various mental healthcare use types and develop inclusive suicide prevention strategies.

## Data Availability

The dataset used in this study, the National Survey on Drug Use and Health, is publicly available at https://www.samhsa.gov/data/data-we-collect/nsduh-national-survey-drug-use-and-health
